# Development of health risk-based metrics for defining a heatwave: a time series study in Brisbane, Australia

**DOI:** 10.1186/1471-2458-14-435

**Published:** 2014-05-09

**Authors:** Shilu Tong, Xiao Yu Wang, Gerry FitzGerald, David McRae, Gerard Neville, Vivienne Tippett, Peter Aitken, Ken Verrall

**Affiliations:** 1 School of Public Health, Institute of Health and Biomedical Innovation, Queensland University of Technology, Brisbane QLD 4000, Australia; 2 Queensland Climate Change Centre of Excellence, Department of Science, Information Technology, Innovation and the Arts, Brisbane, Australia; 3 Environmental Health Branch, Queensland Health, Brisbane, Australia; 4 School of Clinical Sciences, Queensland University of Technology, Brisbane QLD 4000, Australia; 5 Anton Breinl Centre for Public Health and Tropical Medicine, James Cook University, Townsville, Australia; 6 Environmental and Resource Sciences Division, Department of Environment and Resource Management, Brisbane, Australia

**Keywords:** Climate changes, Emergency hospital admissions, Heatwaves, Mean temperature, Mortality, Time series analysis

## Abstract

**Background:**

This study attempted to develop health risk-based metrics for defining a heatwave in Brisbane, Australia.

**Methods:**

Poisson generalised additive model was performed to assess the impact of heatwaves on mortality and emergency hospital admissions (EHAs) in Brisbane.

**Results:**

In general, the higher the intensity and the longer the duration of a heatwave, the greater the health impacts. There was no apparent difference in EHAs risk during different periods of a warm season. However, there was a greater risk for mortality in the 2nd half of a warm season than that in the 1st half. While elderly (≥75 years) were particularly vulnerable to both the EHA and mortality effects of a heatwave, the risk for EHAs also significantly increased for two other age groups (0 – 64 years and 65 – 74 years) during severe heatwaves. Different patterns between cardiorespiratory mortality and EHAs were observed. Based on these findings, we propose the use of a tiered heat warning system based on the health risk of heatwave.

**Conclusions:**

Health risk-based metrics are a useful tool for the development of local heatwave definitions. This tool may have significant implications for the assessment of heatwave-related health consequences and development of heatwave response plans and implementation strategies.

## Background

The health impacts of heatwaves are increasingly recognised and reported [1-4]. It is important to understand the relationship between heatwaves and human health because climate change is likely to increase the frequency, duration, intensity, and geographic extent of heatwaves [5]. Some studies have applied a descriptive approach to estimating excess mortality associated with heatwaves, by comparing the number of deaths between heatwave and non-heatwave days [6,7]. Other studies have used time-series analysis to estimate health risks of hot temperature [8-10]. Recent studies have assessed both the main effect of daily high temperature and added effect of heatwaves (i.e., the impact of extended periods of extreme temperatures) [1,11,12]. However, the findings of these studies have not been consistent. For example, heatwaves or extended periods of extreme temperatures were reported to substantially increase risk compared with that associated with single days of high temperatures [1], but recent studies found that estimated added effect of heatwaves on mortality was much smaller than the main effect of daily high temperature [11-14].

Even though there is no standard definition for heatwaves, a combination of intensity and duration of heat (or extreme high temperatures) has been widely used in previous studies [1,2,4,11,12,15,16]. However, evidence suggests that acclimatization, individual susceptibility, and community characteristics all affect heat-related effects on mortality [1]. This implies that it is difficult, if not impossible, to develop a universal definition of heatwave. Some efforts have been made to explore the best temperature predictor of mortality and an appropriate approach to develop a local definition of heatwave [15,17-19], but, few data are available on the use of health risk-based metrics in the development of a heatwave definition. This study attempted to develop health risk-based metrics to define a heatwave based on the estimation of its effects on mortality and emergency hospital admissions in Brisbane, Australia. Additionally, we evaluated whether the effect estimates varied by heatwave characteristics (intensity, duration, and timing in season) and individual characteristics (age and disease category).

## Methods

This study was conducted in Brisbane, the capital city of Queensland, Australia. It has a typical subtropical climate, with dry and mild winter and humid and warm summer. Its population size at 30 June 2011 was 1,079,392 persons [20].

### Data collection

We obtained daily mortality data for the time period 1 January 1996–30 November 2004, and emergency hospital admissions (EHAs) data between 1 January 1996 and 31 December 2005. Daily data on mortality and EHAs were provided by the Office of Economic and Statistical Research of the Queensland Treasury and the Health Information Centre of Queensland Health, respectively. Mortality data were only obtainable to November 2004 due to the time lag between deaths and their registration by state authorities when we collected these data. Non-external causes (NEC) of mortality and EHAs were categorised according to the International Classification of Diseases (ICD), ninth version (ICD-9) before December 1996 and tenth version (ICD-10) since December 1996 (ICD 9, < 800; and all ICD 10 codes excluding S00-U99 for external causes), cardiovascular diseases (ICD-9, 390–459; ICD-10, I00-I99), and respiratory diseases (ICD-9, 460–519; ICD-10, J00-J99).

Daily data on climatic variables (maximum temperature (°C), minimum temperature (°C) and relative humidity (%)) for the period of January 1996 to December 2005 were obtained from the Australian Bureau of Meteorology. The daily average values of climatic variables were calculated from four monitoring stations located in Brisbane city.

Air pollution data including ambient 24 h average concentrations of particulate matter with diameter less than 10 micrometers (PM_10_) (μg/m^3^), daily maximum 1 hour average nitrogen dioxide (NO_2_) (ppb) and ozone (O_3_) (ppb) were provided by the Queensland Department of Environment and Resource Management (formerly the Queensland Environmental Protection Agency). Daily air pollution concentrations were averaged across seventeen available monitoring stations in Brisbane. When data were missing for a particular monitoring station on a given day (5% of missing data), the observations recorded from the other monitoring stations were used to calculate the daily average values.

### Data analysis

In this study, we used daily mean temperature (calculated using maximum and minimum temperatures) since our previous work suggests that the mean temperature appears to be a reliable indicator of the temperature-health relation [18]. This may be because it was more likely to represent the temperature level across the whole 24 hours. A heatwave was defined as two or more consecutive days with the mean temperature above a certain percentile (e.g., 98th centile of the mean temperature) in the warm season (five months) from the 1st November to the 31st March (next year) during the study period (1996 – 2005) [21,22]. We arranged columns of each mean temperature percentile in the dataset. The binary variable (1 or 0) was entered for heatwave and non-heatwave days, respectively, during the whole study period. We used a relative index (i.e., percentile of temperature) instead of an absolute measure (i.e., degrees in Celsius) for the heatwave definition, as the relation between temperature and health may change over time and space, and therefore, a relative measure (e.g., temperature percentile) may be more appropriate than an absolute one. Relative temperature measures have been increasingly used in recent epidemiological studies [15,21,22]. We divided warm season into two parts (early warm season: 1st Nov. - 15th Jan. (next year) and late warm season: 16th Jan. - 31st Mar.). We just attempted to explore any difference in the health impacts of heatwaves between two different time periods. Poisson generalised additive model (GAM) was used to examine the heatwave effects on NEC mortality and EHAs where adjusting for an array of potential confounders including long-term trends, day of week, relative humidity, PM_10_, NO_2_ and O_3_. Additionally, we assessed the difference in effect estimates by heatwave characteristics (intensity, duration, and timing in season) and individual characteristics (age and disease category) using the same approach. We used linear function for air pollutants, and natural cubic spline for humidity (df = 3) and day of the year (df = 4). We divided the sample into 3 age groups (0 – 64, 65 – 74, and ≥ 75), because we obtained EHA data by four age groups (i.e., 0–14, 15 – 64, 65 – 74, and ≥ 75) due to the reason of confidentiality, and combined the 0 – 14 and the 15 – 64 age groups as there were few records in the 0–14 age group. Mortality data were also matched by these groups. Lagged effects of single lag (lag 0, 1, 2 or 3 days) and cumulative lag (lag 0–1, 0–2 or 0–3 days) were also assessed using the same methods. We used lags (0 to 3) to explore the lag effects of temperature on mortality, because heat effects are usually acute and short-term [23-26]. Relative risks (RRs) and 95% confidence intervals (CIs) were calculated using the GAM model. The “mgcv” package in R software (version 2.14.1) was used to fit the time series GAM.

## Results

The average number of daily deaths and EHAs was 15 and 129, respectively. The average level of daily air pollutants including PM_10_, NO_2_ and O_3_ was 17.4 (μg/m^3^), 13.1 (ppb) and 32.0 (ppb), respectively. The average daily maximum temperature was 28.9°C; minimum temperature was 19.6°C; mean temperature was 24.2°C; and relative humidity was 64.9% (Table 1).

**Table 1:** **Summary of daily health outcomes, air pollutants and climatic variables in Brisbane, Australia, in warm season**^**a **^**(1996 – 2005)**

**Variable**	**Mean**	**SD**	**Min**	**90%**	**95%**	**96%**	**97%**	**98%**	**99%**	**Max**
Deaths	15	4	5	20	21	22	22	23	25	42
EHAs	129	21	71	156	165	166	171	175	181	202
Air pollutant										
PM_10_ (μg/m^3^)	17.4	6.1	6.5	24.2	28.3	29.6	31.4	33.5	39.4	78.6
NO_2_ (ppb)	13.1	4.1	3.6	18.2	20.7	21.6	22.7	23.7	26.4	35.0
O_3_ (ppb)	32.0	11.6	7.7	47.9	53.8	55.8	58.4	61.3	66.9	88.2
Temperature (°C)										
Maximum	28.9	2.5	20.3	32.0	32.9	33.2	33.7	34.2	35.5	41.2
Minimum	19.6	2.6	11.8	22.9	23.8	24.0	24.3	24.6	25.3	27.7
Mean	24.2	2.2	18.1	27.1	28.0	28.1	28.5	28.9	29.9	34.5
Humidity (%)	64.9	11.7	16.0	79.9	83.9	85.9	88.0	89.6	91.5	96.5

90%, 95% to 99% represent the 90th, 95th to 99th centiles, respectively.

*SD* standard deviation, *Min* minimum, *Max* maximum, *EHAs* emergency hospital admissions.

^a^Warm season: 1st Nov. to 31st Mar. (next year).

Table 2 shows the number of heatwave events and days by different centiles of daily mean temperatures according to the heatwave definition (≥2 consecutive days above a certain centile) in warm season and two different parts of warm season (i.e., early and late). Initial analysis indicates that during the study period more intense and longer duration heatwaves were more prevalent during the late warm season. The most severe heatwave occurred during 20 – 22 Feb. 2004, when three consecutive days with a mean temperature above 30°C were recorded in Brisbane. All heatwave events recorded during the study period occurred between the 1 December and the end of February.

**Table 2:** **Heatwave**^**a **^**events and days by percentiles of daily mean temperatures in the warm season**^**b **^**and different parts of warm season (i.e., early and late) in Brisbane, Australia, 1996 - 2005**

**Intensity of percentile**	**Heatwave event (day)**		
	**Warm season**	**Early warm season**^**c**^	**Late warm season**^**d**^
≥99% (29.9°C)	3 (8)	1 (3)	2 (5)
≥98% (28.9°C)	8 (18)	4 (9)	4 (9)
≥97% (28.5°C)	10 (28)	5 (12)	5 (16)
≥96% (28.1°C)	19 (54)	9 (22)	10 (32)
≥95% (28.0°C)	20 (59)	9 (24)	11 (35)
≥90% (27.1°C)	34 (118)	16 (52)	18 (66)

^a^Heatwave defined as ≥ 2 consecutive days above the percentiles of daily mean temperature.

^b^Warm season: 1st Nov. to 31st Mar. (next year).

^c^Early warm season: 1st Nov. to 15th Jan. (next year). ^d^late warm season: 16th Jan. to 31st Mar.

Tables 3 and 4 reveal both the unadjusted and adjusted relative risks of mortality and EHAs in single lag (lag 0, 1, 2 or 3 days) and cumulative lag (lag 0–1, 0–2 or 0–3 days) effects during heatwaves using the different temperature centiles as cut-offs. In general, heatwaves appeared to affect mortality more than EHAs. The higher risk estimates were primarily observed at lags of 1 and 2 days for both mortality and EHAs and then these estimates decreased, which suggests the acute and short term health effects of heatwaves.

**Table 3:** **Relative risk of daily mortality and EHAs for percentile of mean temperature in warm season**^**a **^**(single lag effects)**

**Intensity of percentile**	**Unadjusted RR [95% CI]**		**Adjusted**^**b **^**RR [95% CI]**	
	**Deaths**	**EHAs**	**Deaths**	**EHAs**
Lag 0				
≥99%	1.58 [1.36 - 1.83]	1.25 [1.19 - 1.33]	1.49 [1.28 - 1.73]	1.17 [1.11 - 1.24]
≥98%	1.35 [1.20 - 1.51]	1.20 [1.16 - 1.25]	1.28 [1.14 - 1.43]	1.14 [1.10 - 1.19]
≥97%	1.34 [1.22 - 1.46]	1.16 [1.13 - 1.20]	1.26 [1.15 - 1.39]	1.12 [1.09 - 1.16]
≥96%	1.29 [1.20 - 1.39]	1.14 [1.12 - 1.17]	1.23 [1.14 - 1.33]	1.13 [1.10 - 1.15]
≥95%	1.29 [1.20 - 1.38]	1.14 [1.11 - 1.16]	1.22 [1.14 - 1.32]	1.12 [1.10 - 1.15]
≥90%	1.19 [1.13 - 1.26]	1.07 [1.05 - 1.09]	1.14 [1.08 - 1.20]	1.08 [1.06 - 1.10]
Lag 1				
≥99%	1.76 [1.53 - 2.02]	1.34 [1.27 - 1.42]	1.67 [1.44 - 1.93]	1.28 [1.21 - 1.35]
≥98%	1.39 [1.24 - 1.55]	1.22 [1.17 - 1.27]	1.32 [1.18 - 1.49]	1.16 [1.12 - 1.21]
≥97%	1.38 [1.26 - 1.51]	1.16 [1.13 - 1.20]	1.33 [1.21 - 1.47]	1.12 [1.08 - 1.15]
≥96%	1.26 [1.17 - 1.35]	1.14 [1.12 - 1.17]	1.21 [1.12 - 1.31]	1.12 [1.09 - 1.14]
≥95%	1.25 [1.17 - 1.34]	1.14 [1.11 - 1.16]	1.21 [1.12 - 1.30]	1.12 [1.09 - 1.14]
≥90%	1.15 [1.09 - 1.21]	1.07 [1.05 - 1.09]	1.11 [1.05 - 1.17]	1.08 [1.06 - 1.10]
Lag 2				
≥99%	1.74 [1.52 - 2.01]	1.34 [1.27 - 1.41]	1.68 [1.45 - 1.94]	1.29 [1.22 - 1.36]
≥98%	1.40 [1.25 - 1.56]	1.19 [1.15 - 1.24]	1.36 [1.21 - 1.52]	1.15 [1.11 - 1.20]
≥97%	1.29 [1.17 - 1.41]	1.16 [1.13 - 1.20]	1.24 [1.13 - 1.37]	1.13 [1.09 - 1.17]
≥96%	1.20 [1.12 - 1.29]	1.13 [1.11 - 1.16]	1.17 [1.08 - 1.26]	1.11 [1.09 - 1.14]
≥95%	1.18 [1.10 - 1.26]	1.13 [1.11 - 1.16]	1.14 [1.06 - 1.23]	1.11 [1.08 - 1.13]
≥90%	1.10 [1.04 - 1.16]	1.07 [1.05 - 1.09]	1.07 [1.02 - 1.13]	1.08 [1.06 - 1.10]
Lag 3				
≥99%	1.31 [1.12 - 1.54]	1.22 [1.16 - 1.29]	1.27 [1.08 - 1.49]	1.19 [1.12 - 1.26]
≥98%	1.24 [1.10 - 1.39]	1.14 [1.10 - 1.19]	1.21 [1.07 - 1.36]	1.13 [1.08 - 1.17]
≥97%	1.21 [1.10 - 1.33]	1.12 [1.09 - 1.16]	1.18 [1.07 - 1.30]	1.10 [1.06 - 1.14]
≥96%	1.16 [1.08 - 1.25]	1.11 [1.08 - 1.13]	1.14 [1.06 - 1.23]	1.09 [1.07 - 1.12]
≥95%	1.15 [1.07 - 1.23]	1.10 [1.08 - 1.13]	1.13 [1.05 - 1.22]	1.09 [1.06 - 1.11]
≥90%	1.11 [1.05 - 1.17]	1.05 [1.03 - 1.07]	1.10 [1.04 - 1.16]	1.05 [1.03 - 1.07]

*EHAs* emergency hospital admissions, *RR* relative risk, *CI* confidence interval.

^a^Warm season: 1st Nov. to 31st Mar. (next year).

^b^Confounders including long-term trends, day of week, humidity, PM_10_, NO_2_ and O_3_.

**Table 4:** **Relative risk of daily mortality and EHAs for percentile of mean temperature in warm season**^**a **^**(cumulative lag effects)**

**Intensity of percentile**	**Unadjusted RR [95% CI]**		**Adjusted**^**b **^**RR [95% CI]**	
	**Deaths**	**EHAs**	**Deaths**	**EHAs**
Lags 0-1				
≥99%	1.66 [1.47 - 1.88]	1.27 [1.22 - 1.33]	1.58 [1.39 - 1.79]	1.20 [1.15 - 1.26]
≥98%	1.36 [1.24 - 1.49]	1.20 [1.16 - 1.23]	1.30 [1.18 - 1.43]	1.14 [1.10 - 1.18]
≥97%	1.33 [1.23 - 1.44]	1.14 [1.11 - 1.17]	1.28 [1.18 - 1.39]	1.10 [1.07 - 1.13]
≥96%	1.26 [1.18 - 1.34]	1.13 [1.11 - 1.16]	1.21 [1.13 - 1.30]	1.12 [1.09 - 1.14]
≥95%	1.24 [1.17 - 1.32]	1.13 [1.10 - 1.15]	1.20 [1.12 - 1.28]	1.11 [1.09 - 1.14]
≥90%	1.16 [1.11 - 1.22]	1.07 [1.05 - 1.08]	1.12 [1.06 - 1.17]	1.08 [1.06 - 1.10]
Lags 0-2				
≥99%	1.64 [1.47 - 1.83]	1.26 [1.21 - 1.31]	1.57 [1.40 - 1.76]	1.20 [1.15 - 1.25]
≥98%	1.35 [1.24 - 1.46]	1.18 [1.14 - 1.21]	1.30 [1.19 - 1.42]	1.13 [1.10 - 1.16]
≥97%	1.29 [1.20 - 1.38]	1.14 [1.12 - 1.17]	1.25 [1.16 - 1.35]	1.11 [1.08 - 1.14]
≥96%	1.23 [1.16 - 1.30]	1.13 [1.11 - 1.15]	1.19 [1.12 - 1.27]	1.11 [1.09 - 1.13]
≥95%	1.21 [1.14 - 1.28]	1.12 [1.10 - 1.14]	1.17 [1.10 - 1.25]	1.10 [1.08 - 1.13]
≥90%	1.15 [1.10 - 1.20]	1.07 [1.06 - 1.08]	1.11 [1.06 - 1.16]	1.08 [1.06 - 1.10]
Lags 0-3				
≥99%	1.53 [1.38 - 1.70]	1.25 [1.20 - 1.30]	1.47 [1.32 - 1.64]	1.20 [1.15 - 1.24]
≥98%	1.31 [1.21 - 1.42]	1.17 [1.14 - 1.20]	1.27 [1.17 - 1.38]	1.13 [1.10 - 1.16]
≥97%	1.25 [1.17 - 1.33]	1.14 [1.11 - 1.16]	1.21 [1.13 - 1.30]	1.11 [1.08 - 1.13]
≥96%	1.20 [1.14 - 1.27]	1.12 [1.10 - 1.14]	1.17 [1.11 - 1.24]	1.11 [1.09 - 1.13]
≥95%	1.19 [1.13 - 1.25]	1.12 [1.10 - 1.13]	1.16 [1.09 - 1.22]	1.10 [1.08 - 1.12]
≥90%	1.13 [1.09 - 1.18]	1.07 [1.06 - 1.08]	1.10 [1.05 - 1.15]	1.08 [1.06 - 1.09]

*EHAs* emergency hospital admissions, *RR* relative risk, *CI* confidence interval.

^a^Warm season: 1st Nov. to 31st Mar. (next year).

^b^Confounders including long-term trends, day of week, humidity, PM_10_, NO_2_ and O_3_.

Tables 5 and 6 reveal both unadjusted and adjusted relative risks for mortality and EHAs at lag 0 (current day) during heatwaves using the different temperature centiles as the cut-offs. When investigating any difference in the heatwave effects between the early and late of warm season, we found that the impact of heatwaves on mortality and EHAs seemed to be stronger in the second part of the warm season than the first (Table 5).

**Table 5:** **Relative risk of daily mortality and EHAs for percentile of mean temperature in early and late parts of warm season**^**a**^

**Intensity of percentile**	**Unadjusted RR [95% CI]**		**Adjusted**^**b **^**RR [95% CI]**	
	**Deaths**	**EHAs**	**Deaths**	**EHAs**
Early warm season^c^				
≥99%	1.10 [0.82 - 1.46]	**1.12 [1.02 - 1.22]**	1.04 [0.78 - 1.40]	1.08 [0.98 - 1.18]
≥98%	**1.24 [1.03 - 1.48]**	**1.17 [1.11 - 1.24]**	**1.19 [1.00 - 1.43]**	**1.14 [1.08 - 1.20]**
≥97%	1.12 [0.96 - 1.31]	**1.14 [1.09 - 1.20]**	1.08 [0.91 - 1.27]	**1.11 [1.06 - 1.17]**
≥96%	**1.18 [1.05 - 1.33]**	**1.13 [1.09 - 1.17]**	**1.14 [1.01 - 1.29]**	**1.11 [1.07 - 1.15]**
≥95%	**1.18 [1.05 - 1.33]**	**1.14 [1.10 - 1.18]**	**1.14 [1.01 - 1.29]**	**1.12 [1.08 - 1.16]**
≥90%	**1.09 [1.00 - 1.18]**	**1.06 [1.04 - 1.09]**	1.05 [0.97 - 1.15]	**1.07 [1.04 - 1.10]**
Late warm season^d^				
≥99%	**1.88 [1.58 - 2.24]**	**1.35 [1.26 - 1.44]**	**1.71 [1.42 - 2.07]**	**1.20 [1.11 - 1.29]**
≥98%	**1.43 [1.24 - 1.66]**	**1.23 [1.17 - 1.30]**	**1.29 [1.11 - 1.51]**	**1.14 [1.08 - 1.20]**
≥97%	**1.48 [1.33 - 1.65]**	**1.19 [1.14 - 1.24]**	**1.35 [1.20 - 1.52]**	**1.13 [1.08 - 1.18]**
≥96%	**1.37 [1.25 - 1.50]**	**1.16 [1.12 - 1.19]**	**1.26 [1.15 - 1.39]**	**1.14 [1.10 - 1.17]**
≥95%	**1.35 [1.24 - 1.47]**	**1.14 [1.11 - 1.17]**	**1.24 [1.13 - 1.37]**	**1.13 [1.09 - 1.16]**
≥90%	**1.27 [1.19 - 1.36]**	**1.08 [1.06 - 1.11]**	**1.19 [1.10 - 1.28]**	**1.10 [1.07 - 1.12]**

*EHAs* emergency hospital admissions, *RR* relative risk, *CI* confidence interval.

^a^Warm season: 1st Nov. to 31st Mar. (next year).

^b^Confounders including long-term trends, day of week, humidity, PM_10_, NO_2_ and O_3_.

^c^Early warm season: 1st Nov. to 15th Jan. (next year).

^d^Late warm season: 16th Jan. to 31st Mar.

Bold typeface indicates statistical significance at p<0.05.

**Table 6:** **Relative risk of daily mortality and EHAs for percentile of mean temperature in warm season**^**a **^**by diseases and age groups**

**Intensity of percentile**	**Unadjusted RR [95% CI]**		**Adjusted**^**b **^**RR [95% CI]**	
	**Deaths**	**EHAs**	**Deaths**	**EHAs**
Respiratory				
≥99%	**1.70 [1.04 - 2.80]**	**1.35 [1.16 - 1.58]**	1.34 [0.80 - 2.23]	**1.30 [1.11 - 1.52]**
≥98%	**1.75 [1.24 - 2.48]**	**1.19 [1.07 - 1.33]**	**1.49 [1.04 - 2.14]**	**1.14 [1.02 - 1.28]**
≥97%	**1.55 [1.15 - 2.08]**	**1.13 [1.03 - 1.24]**	**1.37 [1.00 - 1.87]**	**1.10 [1.00 - 1.21]**
≥96%	**1.45 [1.15 - 1.83]**	**1.14 [1.07 - 1.22]**	**1.34 [1.05 - 1.72]**	**1.12 [1.04 - 1.20]**
≥95%	**1.42 [1.13 - 1.79]**	**1.14 [1.07 - 1.21]**	**1.32 [1.04 - 1.69]**	**1.11 [1.04 - 1.19]**
≥90%	**1.33 [1.11 - 1.58]**	**1.08 [1.03 - 1.13]**	**1.31 [1.09 - 1.58]**	**1.07 [1.01 - 1.12]**
Cardiovascular				
≥99%	**1.90 [1.56 - 2.33]**	1.04 [0.90 - 1.20]	**1.81 [1.47 - 2.23]**	1.04 [0.89 - 1.20]
≥98%	**1.46 [1.24 - 1.72]**	1.07 [0.98 - 1.18]	**1.38 [1.16 - 1.63]**	1.06 [0.97 - 1.17]
≥97%	**1.50 [1.32 - 1.71]**	1.00 [0.92 - 1.08]	**1.41 [1.23 - 1.62]**	0.99 [0.92 - 1.08]
≥96%	**1.36 [1.23 - 1.51]**	0.99 [0.94 - 1.05]	**1.28 [1.15 - 1.43]**	0.99 [0.93 - 1.05]
≥95%	**1.37 [1.24 - 1.51]**	1.00 [0.95 - 1.06]	**1.29 [1.16 - 1.43]**	1.00 [0.94 - 1.06]
≥90%	**1.23 [1.14 - 1.33]**	0.98 [0.94 - 1.02]	**1.16 [1.07 - 1.26]**	0.98 [0.94 - 1.02]
0 - 64 age group				
≥99%	**1.49 [1.03 - 2.15]**	**1.13 [1.05 - 1.22]**	**1.49 [1.02 - 2.17]**	1.06 [0.98 - 1.14]
≥98%	1.28 [0.97 - 1.69]	**1.14 [1.08 - 1.19]**	1.26 [0.95 - 1.68]	**1.08 [1.03 - 1.14]**
≥97%	1.19 [0.94 - 1.50]	**1.11 [1.07 - 1.16]**	1.16 [0.92 - 1.48]	**1.07 [1.03 - 1.12]**
≥96%	**1.20 [1.00 - 1.43]**	**1.12 [1.08 - 1.15]**	1.17 [0.97 - 1.41]	**1.10 [1.07 - 1.13]**
≥95%	**1.21 [1.02 - 1.43]**	**1.11 [1.08 - 1.14]**	1.18 [0.98 - 1.41]	**1.10 [1.06 - 1.13]**
≥90%	1.13 [0.99 - 1.28]	**1.06 [1.03 - 1.08]**	1.09 [0.95 - 1.24]	**1.07 [1.04 - 1.09]**
65 - 74 age group				
≥99%	1.38 [0.96 - 1.99]	**1.21 [1.04 - 1.41]**	1.41 [0.96 - 2.05]	**1.24 [1.06 - 1.45]**
≥98%	1.11 [0.83 - 1.48]	1.10 [0.99 - 1.22]	1.10 [0.82 - 1.49]	1.11 [0.99 - 1.24]
≥97%	1.13 [0.90 - 1.41]	**1.12 [1.03 - 1.22]**	1.09 [0.86 - 1.39]	**1.14 [1.04 - 1.24]**
≥96%	1.13 [0.95 - 1.35]	**1.08 [1.01 - 1.15]**	1.09 [0.91 - 1.31]	**1.09 [1.02 - 1.16]**
≥95%	1.11 [0.93 - 1.32]	**1.07 [1.01 - 1.14]**	1.06 [0.89 - 1.27]	**1.08 [1.01 - 1.15]**
≥90%	1.01 [0.89 - 1.15]	1.02 [0.97 - 1.07]	0.94 [0.82 - 1.08]	1.02 [0.97 - 1.07]
75 and over age group				
≥99%	**1.66 [1.39 - 1.98]**	**1.55 [1.41 - 1.71]**	**1.51 [1.26 - 1.81]**	**1.39 [1.26 - 1.54]**
≥98%	**1.43 [1.25 - 1.64]**	**1.40 [1.31 - 1.50]**	**1.33 [1.15 - 1.53]**	**1.29 [1.20 - 1.39]**
≥97%	**1.43 [1.29 - 1.60]**	**1.31 [1.24 - 1.39]**	**1.33 [1.19 - 1.49]**	**1.23 [1.16 - 1.31]**
≥96%	**1.36 [1.25 - 1.48]**	**1.25 [1.19 - 1.30]**	**1.29 [1.18 - 1.41]**	**1.21 [1.15 - 1.26]**
≥95%	**1.36 [1.25 - 1.47]**	**1.24 [1.19 - 1.29]**	**1.28 [1.17 - 1.40]**	**1.21 [1.16 - 1.26]**
≥90%	**1.26 [1.18 - 1.34]**	**1.13 [1.09 - 1.16]**	**1.21 [1.13 - 1.29]**	**1.14 [1.10 - 1.17]**

EHAs, emergency hospital admissions; RR, relative risk; CI, confidence interval.

^a^Warm season: 1st Nov. to 31st Mar. (next year).

^b^Confounders including long-term trends, day of week, humidity, PM_10_, NO_2_ and O_3_.

Bold typeface indicates statistical significance at p<0.05.

Table 6 indicates that there was a statistically significant increase in both cardiovascular and respiratory mortality during heatwaves at almost all centile cut-offs. Results also show a statistically significant increase in EHAs for respiratory diseases, but not for cardiovascular diseases. The elderly group (≥75) appeared to be most vulnerable to heatwaves for both mortality and EHAs. There was also increased risk in other age groups during heatwaves, particularly for EHAs.

There was a statistically significant increased health risk if the mean temperature exceeded the historical 90th, 95th or 98th centile for at least two, three and four consecutive days after adjustment for confounders including long-term trends, day of week, relative humidity, PM_10_, NO_2_ and O_3_ (Table 7). The historical 90th, 95th and 98th centiles of the mean temperature were 27.1, 28.0 and 28.9°C, respectively. There were no such heatwave events in Brisbane if we defined a heatwave as 4 consecutive days with mean temperature above the 98th centile.

**Table 7:** **Relative risk of daily mortality and EHAs for percentile of mean temperature in warm season**^**a **^**under different heatwave definitions**

**Intensity of percentile**	**RR [95% CI]**^**b**^		**RR [95% CI]**^**c**^		**RR [95% CI]**^**d**^	
	**Deaths**	**EHAs**	**Deaths**	**EHAs**	**Deaths**	**EHAs**
Lag 0						
≥98%	1.28 [1.14 - 1.43]	1.14 [1.10 - 1.19]	1.30 [1.08 - 1.56]	1.21 [1.14 - 1.29]	n/a	n/a
≥95%	1.22 [1.14 - 1.32]	1.12 [1.10 - 1.15]	1.27 [1.17 - 1.38]	1.15 [1.12 - 1.18]	1.19 [1.07 - 1.32]	1.15 [1.11 - 1.19]
≥90%	1.14 [1.08 - 1.20]	1.08 [1.06 - 1.10]	1.15 [1.08 - 1.22]	1.08 [1.05 - 1.10]	1.19 [1.11 - 1.27]	1.08 [1.06 - 1.10]
Lag 1						
≥98%	1.33 [1.18 - 1.49]	1.16 [1.11 - 1.20]	1.35 [1.12 - 1.62]	1.27 [1.19 - 1.35]	n/a	n/a
≥95%	1.20 [1.12 - 1.30]	1.12 [1.09 - 1.14]	1.32 [1.21 - 1.43]	1.15 [1.12 - 1.18]	1.22 [1.10 - 1.35]	1.15 [1.11 - 1.19]
≥90%	1.11 [1.05 - 1.17]	1.08 [1.06 - 1.09]	1.17 [1.10 - 1.24]	1.07 [1.05 - 1.09]	1.21 [1.13 - 1.29]	1.07 [1.05 - 1.10]
Lag 2						
≥98%	1.36 [1.21 - 1.52]	1.15 [1.11 - 1.20]	1.48 [1.24 - 1.76]	1.33 [1.25 - 1.42]	n/a	n/a
≥95%	1.14 [1.06 - 1.23]	1.11 [1.08 - 1.13]	1.22 [1.13 - 1.33]	1.15 [1.12 - 1.18]	1.22 [1.10 - 1.35]	1.13 [1.09 - 1.17]
≥90%	1.07 [1.02 - 1.13]	1.08 [1.06 - 1.10]	1.13 [1.06 - 1.20]	1.07 [1.05 - 1.09]	1.17 [1.10 - 1.25]	1.06 [1.04 - 1.08]
Lags 0 - 2						
≥98%	1.30 [1.19 - 1.42]	1.13 [1.10 - 1.16]	1.30 [1.12 - 1.50]	1.24 [1.18 - 1.30]	n/a	n/a
≥95%	1.17 [1.10 - 1.25]	1.10 [1.08 - 1.13]	1.24 [1.15 - 1.33]	1.13 [1.11 - 1.16]	1.19 [1.09 - 1.30]	1.13 [1.10 - 1.17]
≥90%	1.11 [1.06 - 1.16]	1.08 [1.06 - 1.10]	1.15 [1.09 - 1.22]	1.06 [1.05 - 1.08]	1.19 [1.13 - 1.27]	1.07 [1.05 - 1.09]

*EHAs* emergency hospital admissions, *RR* relative risk, *CI* confidence interval.

^a^Warm season: 1st Nov. to 31st Mar. (next year).

^b^Heatwave defined as ≥ 2 consecutive days above the percentiles of daily mean temperature.

^c^Heatwave defined as ≥ 3 consecutive days above the percentiles of daily mean temperature.

^d^Heatwave defined as ≥ 4 consecutive days above the percentiles of daily mean temperature.

**Figure 1:**
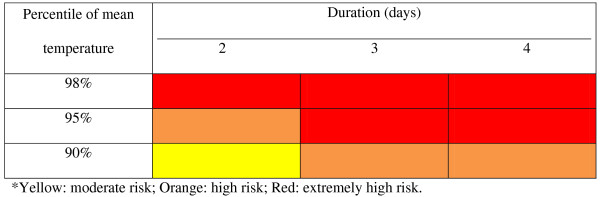
Health risk-based metrics based on the impacts of heat on mortality and EHAs.

Based on the overall results, we developed preliminary health risk-based metrics to define a heatwave at the local level (Figure 1). Thus, we propose the use of a tiered heat warning system based on the likely severity of heatwave: yellow (≥90th centile of historical mean temperature for two or more consecutive days: moderate risk); orange (≥95th centile of historical mean temperature for two or more consecutive days: high risk); and red warning (≥98th centile of historical mean temperature for two or more consecutive days: extreme high risk). At such times, vulnerable groups (e.g., children, elderly, people with disability, residents with chronic disease, economically disadvantaged and/or isolated people) should be particularly protected.

## Discussion

This study investigated the potential to develop local temperature/health metrics based on the estimation of the effects of heatwaves on mortality and emergency hospital admissions, and evaluated whether the effect estimates varied by heatwave characteristics (intensity, duration, and timing in season) and individual characteristics (age and disease category) in Brisbane, Australia.

The results of this study show that there were consistent and significantly increased risks of death and EHAs during heatwaves even using different temperature cut-offs. In general, the more intense and longer duration heatwave events, the greater health impacts, which are consistent with previous findings [11,22,27]. Brisbane has a humid subtropical climate condition where the average relative humidity was 64.9% in warm seasons during the study period. However, it had been hot and dry during the heatwave periods. For example the average relative humidity was 61.9% and 54.2% when heatwave defined as two or more consecutive days with mean temperature above 95th and 98th centile, respectively. Based on the data, heatwave events also appeared to affect mortality more than EHAs, which is supported by the literature [28-30]. A possible explanation for the mismatch in patterns of mortality and hospital admissions during hot weather is that many heat-related deaths occur in isolated people before they come to medical attention [28]. For example, many of the deaths in the Chicago heatwaves were of people living alone or having limited social contact [7,31]. However, we found that the impact of heatwaves on mortality and EHAs seemed to be stronger in the second part of the warm season than the first part (Table 5), which is in contrast to the previous studies [11,22]. When investigating this difference, we found that the more intense and longer duration heatwave events in Brisbane occurred more frequently during the second part of the warm season than the first part (Table 2). In addition, mean temperature and relative humidity levels during the second period were 1 to 2°C and 3 to 4% higher than the first period (data not shown). This may cause people to feel more uncomfortable in the late warm season than the early season. It may also include a variation in susceptible population throughout the summer with people coming and going from Brisbane during the holiday season. In an assessment of the health impact of heatwaves, it is therefore desirable to adopt an integrated approach which takes the intensity, duration and timing into account systematically and dynamically.

The elderly group (≥75) was most vulnerable to heatwaves for both mortality and EHAs which is similar across the warm season (Table 6). This finding is consistent with most previous studies [9,12]. Increased platelet and red cell counts, blood viscosity, and plasma cholesterol levels were reported during heat stress, which may explain the increased mortality from arterial thrombosis in hot weather [32]. Heat-related mortality substantially increases in older people, which may be due to their poor tolerance to temperature variation and compromised ability to maintain core body temperature. This is reflected by research reporting that thermal sensitivity decreases with advancing age [33]. A recent study observed that the elderly had poor thermoregulatory responses to high temperatures because of hormonal changes with age [34]. Reduced thermoregulatory responses and less sensitive thermal perception in the elderly may blunt thermoregulatory behaviour during heat stress and facilitate the occurrence of hyperthermia [35]. On the other hand, younger people exercising outdoors in high temperatures may also experience increased heat-related morbidity or mortality.

The results of this study highlight a statistically significant increase in cardiovascular and respiratory mortality and EHAs for respiratory diseases during heatwaves, but no significant increase in EHAs for cardiovascular diseases. Our findings are broadly consistent with previous studies [28-30]. The hypothesis for the difference in cardiovascular mortality and hospital admissions is that cardiovascular deaths usually occur rapidly before they reach a hospital [36].

Based on our findings, we propose the use of a tiered heat warning system primarily based on the likely severity of heatwave: yellow (≥90th centile of historical mean temperature for two or more consecutive days: moderate risk); orange (≥95th centile of historical mean temperature for two or more consecutive days: high risk); and red warning (≥98th centile of historical mean temperature for two or more consecutive days: extreme high risk). Vulnerable groups (e.g., children, elderly, people with disability, residents with chronic disease, economically disadvantaged and/or isolated people) should be particularly protected. As a number of studies have demonstrated that heat-related deaths can happen rapidly [3,22,27,28,30], the heat warning system should be activated well before a heatwave occurs. As both climate predictions (e.g., El Niňo events) and weekly weather forecasting are readily available for most cities, it would be feasible to trigger a heat warning system a week before a heatwave event is anticipated. If the proposed tiered heat warning system is confirmed by further research in different populations, it may have significant implications in reducing and preventing heat-related deaths and potentially mitigate likely health impacts of anthropogenic climate change.

Over the past several years, a number of heat watch–warning systems have been implemented in different cities across the world, based on local health response to past weather conditions [37,38]. For example, after the 2003 heatwave, a heat watch warming system was developed and implemented in France which was based on retrospective analyses of mortality and meteorological data in fourteen pilot cities [38]. However, they used the same percentile of temperature to trigger the national action plan even though the health effects of heatwaves were apparently heterogeneous. To our knowledge, there is no heatwave plan for the whole Australia up to now. Different heatwave definitions were used in previous Australian studies [15,39,40]. Therefore, it is an urgent need to develop an appropriate approach to define a heatwave at a local and/or regional level.

This study has three key strengths. As this study attempted to develop health risk-based metrics for defining a heatwave event at a local level particularly in a subtropical climate, there may be positive implications for the planning and implementation of climate risk management policies. Secondly, mortality and emergency hospital admissions were jointly considered in the assessment of heatwave related health risks. Finally, we were able to include a range of confounding variables in the modelling process, including long-term trends, day of week, relative humidity, and air pollution_._

Two major weaknesses of this study must also be acknowledged. Only one city was considered in this assessment. However, the idea of defining a heatwave using readily available empirical data may inspire further research in this field. The second weakness was that aggregated data were used in assessing the effect of heatwaves on mortality and EHAs. Detailed spatial analysis might give a more precise estimate of the health impacts of a heatwave.

## Conclusions

Health risk-based metrics are a useful tool for the development of a clearly defined definition of a heatwave event. This tool has the potential to be applied in the examination of health risks of climate variability and change and the establishment of heat warning systems. As the relation between temperature and health may change over time and space, it needs to be evaluated in an appropriate way. Nevertheless, the methodology developed in this study may have significant implications for the assessment of heatwave-related health consequences and development of heatwave response plans and implementation strategies.

## Abbreviations

CI: Confidence interval; EHAs: Emergency hospital admissions; GAM: Generalised additive model; ICD: International classification of diseases; NEC: Non-external causes; NO2: Nitrogen dioxide; O3: Ozone; PM10: Particulate matter with diameter less than 10 μm; RR: Relative risk.

## Competing interests

The authors declare that they have no competing interest.

## Authors’ contributions

ST, GF and PA contributed to the design and obtain funding for the study. ST and XW wrote the first draft of the article. ST co-ordinated the study conduct. XW conducted the data analyses. All authors critically reviewed and approved the manuscript for publication.

## Pre-publication history

The pre-publication history for this paper can be accessed here:

http://www.biomedcentral.com/1471-2458/14/435/prepub
